# A New Outlook on Mental Illnesses: Glial Involvement Beyond the Glue

**DOI:** 10.3389/fncel.2015.00468

**Published:** 2015-12-16

**Authors:** Maha Elsayed, Pierre J. Magistretti

**Affiliations:** ^1^Laboratory of Neuroenergetics and Cellular Dynamics, Brain Mind Institute, Ecole Polytechnique Fédérale de LausanneLausanne, Switzerland; ^2^Division of Biological and Environmental Sciences and Engineering, King Abdullah University of Science and TechnologyThuwal, Saudi Arabia; ^3^Department of Psychiatry, Center for Psychiatric Neurosciences, University of LausanneLausanne, Switzerland

**Keywords:** psychiatric disorder, glia, astrocyte, oligodendrocyte, microglia, NG2 glia, mood, cognition

## Abstract

Mental illnesses have long been perceived as the exclusive consequence of abnormalities in neuronal functioning. Until recently, the role of glial cells in the pathophysiology of mental diseases has largely been overlooked. However recently, multiple lines of evidence suggest more diverse and significant functions of glia with behavior-altering effects. The newly ascribed roles of astrocytes, oligodendrocytes and microglia have led to their examination in brain pathology and mental illnesses. Indeed, abnormalities in glial function, structure and density have been observed in postmortem brain studies of subjects diagnosed with mental illnesses. In this review, we discuss the newly identified functions of glia and highlight the findings of glial abnormalities in psychiatric disorders. We discuss these preclinical and clinical findings implicating the involvement of glial cells in mental illnesses with the perspective that these cells may represent a new target for treatment.

## Introduction

Treatment of mental illnesses dates back to ancient times where imprisonment and confinement to chains were the mode of action to control what was perceived as influences of witchcraft and supernatural forces. With the introduction of Hippocratic medicine back in the 4th century B.C., mental illness had a physical attribute and the cause was linked to humoral imbalances. Though, the idea of demons and supernatural forces still persisted. Towards the end of the 18th century, the idea of mental illness as a disease of the mind rather than the body began to develop and it was towards the mid-19th century when it became viewed as a disease of the brain. Though, the term mental was coined to it till this day, mainly due to the lack of cerebral pathology at macroscopic and microscopic levels at the time (Kendell, 2001).

In the 1950s, psychopharmacology emerged. Following serendipitous clinical observations, chlorpromazine (dopamine antagonist) and iproniazid (monoamine oxidase inhibitor) were observed to have antipsychotic and antidepressant effects respectively (Deverteuil and Lehmann, 1958; Ban, 2007; Nestler and Hyman, 2010). These observations paved the way to the development of current psychotropic drugs whose pharmacology is essentially based on monoamine neurotransmission. Despite the availability of these psychoactive medicines, there remains however an increasing prevalence, undeniable disability, economic and social burden (Hyman, 2008). The reason for the lack of success is that these psychotherapeutic drugs were not founded on real evidence of underlying pathology. Instead, the reverse engineering of these drugs lead to the identification of molecular targets that are possibly not the actual culprit. With the emergence of *in vivo* brain imaging techniques and improvement in the methods of microscopy, immunocytochemistry and stereology, interest in re-examining cerebral pathology at the macro- and micro-scopic level ensued (Rajkowska et al., 1999). The microscopic approach has notably brought glial cells to light with newly identified functions. With access to the right tools, findings of glia pathology in psychiatric disorders began to surface (Di Benedetto and Rupprecht, 2013). In this review, we will introduce the different types and functions of glia and then discuss findings implicating their involvement in the different types of mental illnesses.

## Glia in Brain Function and Health

While the legacy of the last century of research in psychiatry has centered on deciphering the role of neuronal systems in brain functions in health and disease, little attention has been paid to non-neuronal cells. Glial cells in fact outnumber neurons in several areas of the human brain (Kandel, 2000; Pelvig et al., 2008; Azevedo et al., 2009; Herculano-Houzel, 2011). Interestingly enough, this ratio is decreased in rodents (Nedergaard et al., 2003; Rajkowska and Miguel-Hidalgo, 2007; Herculano-Houzel, 2011) indicating that increased glial densities is associated with higher brain functions. The term neuroglia was initially coined by the German anatomist Rudolf Virchow in 1856 to refer to a scaffolding material. Traditionally seen as silent supportive cells, growing evidence suggest a more dynamic and active function. Glial cells provide a source of metabolic energy and growth/neurotrophic factors, are involved in regulating synaptic plasticity, modulating neuronal excitability, neurotransmitter modulation/reuptake and relay of information, among other functions. In short, they have emerged to be important players that alter neuronal state and connectivity.

Based on lineages, there are two types of Central Nervous System (CNS) glia: macroglia and microglia. Macroglia (astrocyte, oligodendrocyte) arise from ectoderm while microglia originate from monocyte-macrophage lineage (Ventura and Goldman, 2006; Rajkowska and Miguel-Hidalgo, 2007). Each type has a specialized function and a unique morphology (Ventura and Goldman, 2006; Rajkowska and Miguel-Hidalgo, 2007). While oligodendrocytes and microglia were long thought to have specialized functions, astrocytes proved to be the most complex and functionally diverse.

### Astrocytes

The term astrocyte was initially described by Von Lenhossek in 1893 based on its star-like morphology. It turns out that astrocytes are quite heterogeneous in cell morphology, a fact that also reflects inherent functional specialization. Astrocytes can be categorized into at least five different types: (1) white matter astrocytes which take on a star shape; (2) gray matter astrocytes, which have a less complex shape; (3) ependymal astrocytes, which are stained positive for a marker of astrocytes, GFAP, and are found in the stem cell niches of the brain; (4) radial glia found within ventricular zone which originally provide a scaffold for migrating neurons during brain development; and (5) perivascular, also GFAP+, whose end-feet are in close proximity to blood vessels (Claycomb et al., 2013). Novel discoveries on the diverse functions of astrocytes have challenged the long-time held dogma that astrocytes are merely passive cells. From an evolutionary point of view, the ratio of astrocytes to neurons and the morphology of astrocytes increase with the complexity of brain functions (Oberheim et al., 2009; Pereira and Furlan, 2010; Herculano-Houzel, 2011). The diversity of astrocytic roles are discussed below and range from local modulation of information processing within a synapse to brain large-scale integrative functions, and extend to interactions with the vasculature system and the immune system. Some of these functions support its involvement in cognitive and mood functions and the ones pertinent to psychiatric illnesses are discussed below.

#### Neurovascular Unit

Astrocytes form a bridging gap, coupling the vasculature system with neuronal circuits. The surface of intraparenchymal capillaries is covered at 99% by astrocytic end-feet (Kacem et al., 1998). Astrocytic end feet wrap around the endothelium of blood vessels and via this contact, they can influence cerebral blood flow (Takano et al., 2006; Magistretti and Allaman, 2015) and control the transport of substances in and out of the brain to ensure proper brain homeostasis (Abbott et al., 2006).

#### Metabolic Coupling

Astrocytes have been shown to support neurons metabolically. Astrocytes express glucose transporters of the GLUT1 type along their astrocytic end feet (Allaman and Magistretti, 2013). Upon increased neuronal activity and glutamate reuptake by astrocyte-specific glutamate transporters, a sequence of events is triggered resulting in the uptake of glucose from blood vessels and erobic glycolysis, a process also known as the Astrocyte Neuron Lactate Shuttle (for review, see Magistretti and Allaman, 2015). With Lactate being the end product, it is released into the interstitial space for neuronal uptake (Walz and Mukerji, 1988; Pellerin and Magistretti, 1994; Chuquet et al., 2010). Furthermore, astrocytes are the only cells to store energy in the form of glycogen in the adult brain. It was shown that this energy reserve can be mobilized by various neuroactive signals such as noradrenaline and vasoactive intestinal peptide (Magistretti et al., 1981; Sorg and Magistretti, 1991). Thus, there is an interplay between energy metabolism and neuronal activity with astrocytes being the mediators. Lactate released by astrocytes has been shown to exert additional important physiological functions such as induction of neuroplasticity and taking part in higher cognitive functions such as learning and memory (Suzuki et al., 2011; Yang et al., 2014).

#### Tripartite Synapse, Gliotransmission and Synaptic Function

Astrocytes express a range of receptors and ion channels that are similarly expressed in neurons (Verkhratsky et al., 1998). At most glutamatergic central synapses, the extremity of protoplasmic astrocyte process wraps the synaptic cleft, and communicates with pre- and post-synaptic neurons, forming what is called a tripartite synapse (Araque et al., 1999; Bezzi et al., 2001). At those processes, they express glutamate transporters responsible for glutamate re-uptake and clearance from the synaptic cleft. With this feature, astrocytes can prevent the excitotoxic build-up of glutamate concentrations, and hence provide a form of neuroprotection (Choi, 1987; Rothstein et al., 1996; Tanaka et al., 1997). Furthermore, emerging data suggest that astrocytes are excitable cells able to release transmitters and thus regulate synaptic function. Some of the gliotransmitters released by astrocytes include ATP, D-serine, adenosine, glutamate and cytokines (Volterra and Meldolesi, 2005). Some of these gliotransmitters are involved in modulating synaptic function. For example, D-serine is one of the required coactivators of NMDA receptors at the glycine site. It is three times more potent than glycine (Miyazaki et al., 1999); both D-serine and glycine are released by astrocytes, hence enabling these cells to regulate N-Methyl-D-aspartate (NMDA) receptor activity (Schell et al., 1995; Wolosker et al., 1999a,b). To add another layer of complexity and heterogeneity of astrocyte specializations, distinct population of astrocytes contain exocytosis machinery such as vesicular glutamate transporter (vGluT) and are capable of initiating vesicular release of glutamate upon activation (Bezzi et al., 2004; Kreft et al., 2004; Montana et al., 2004; Zhang et al., 2004; Jourdain et al., 2007).

#### Neurotrophic Function

Astrocytes can synthesize and release many neurotrophic factors such as fibroblast growth factor 2 (FGF2; Gonzalez et al., 1995), brain-derived neurotrophic factor (BDNF; Jean et al., 2008) and other growth factors involved in modulating synaptic transmission and plasticity (Levine et al., 1995; Lo, 1995; Zechel et al., 2010). These growth factors can ultimately impact cognition and mood associated behavior (Graham and Richardson, 2011; Elsayed et al., 2012; Quesseveur et al., 2013).

#### Response to Injury and Pathogen

In response to injury, astrocytes become reactive, change their morphology and pattern of gene expression. They can also be induced to express major histocompatibility complex antigen to identify pathogen, modify Blood Brain Barrier permeability and secrete various cytokines to attract immune cells from the blood circulation (Sparacio et al., 1992; Farina et al., 2007; Burda and Sofroniew, 2014).

#### Gap Junctions

The complexity of astrocytes is further enhanced by the expression of connexins which form gap junctions (Giaume et al., 2005; Orthmann-Murphy et al., 2008). This feature allows the formation of a syncytium whereby astroglia communicates intercellularly. Gap junction coupling is not static and is modulated by a number of signaling pathways (Anders et al., 2014). Mainly, sensory, cognitive and emotional patterns transmitted from spatially distributed neuronal and glial populations can result in the activation of astroglial calcium waves that can be transmitted across the astrocytic syncytium (Pereira and Furlan, 2010).

### Oligodendrocytes

Oligodendrocytes are smaller and less branched than astrocytes (Fawcett, 1994). Similarly to astrocytes, there are two types of oligodendrocyte residents in the cortex: (1) perineuronal oligodendrocytes, which are located in the gray matter and (2) interfascicular oligodendrocytes which are found in the white matter (Rajkowska and Miguel-Hidalgo, 2007). Myelin formation has been the classical function attributed to oligodendrocytes with the function of insulating axons hence enabling faster conduction speed of action of potential. White and gray matter myelinations are exceptionally high in humans when compared to other species including primates (Zhang and Sejnowski, 2000; Miller et al., 2012) pointing to a higher structural connectivity as part of an evolutionary mechanism.

All this comes at a high energetic cost, with a large proportion of brain energy metabolites being directed towards creating and supporting myelination along with maintenance of transmembrane ionic gradients to sustain excitability (Connor and Menzies, 1996; Attwell and Laughlin, 2001; Sanchez-Abarca et al., 2001; Alle et al., 2009; Rinholm et al., 2011).

While initially thought as static components of the nervous system, recent studies suggest that myelin formation by oligodendrocytes is a highly dynamic processes influenced by neuronal activity (Ishibashi et al., 2006), learning (Bengtsson et al., 2005) and environmental input (Markham and Greenough, 2004). Moreover, myelination is not restricted to a developmental program but can occur through adulthood suggesting contribution to brain plasticity (De Hoz and Simons, 2015). By enhancing speed and efficiency of action potential transmission, myelination enables synchronization of neural networks, which underlie the basis of our cognitive and behavioral repertoires and hence making this process a vital one for brain functioning (Haroutunian et al., 2014).

In addition to myelin formation, oligodendrocytes also express growth factors (Byravan et al., 1994), gap junctions (Orthmann-Murphy et al., 2008) and can supply energy in the form of Lactate to support axonal function (Funfschilling et al., 2012; Lee et al., 2012). Furthermore, they express glutamate receptors and are thus a target of neurotransmitters and glutamate excitotoxicity (Matute, 2006).

### NG2-Glia

The notion that the adult brain is a static organ has been disputed over at least the past 30 years. In fact, the brain is a dynamic organ with neuronal and non-neuronal cells undergoing cellular plastic events regulated by endogenous and exogenous cues (Dong and Greenough, 2004). It is accepted now that cell proliferation, one aspect of cellular plasticity, occurs during brain development and continues into adulthood. The areas and rates of cell proliferation vary depending on the cell type in question (i.e., neurons or glia), or on the conditions surrounding these cells. In healthy conditions, gliogenesis is a slow-turnover process that occurs in the white and gray matter of the adult brain. It involves the proliferation of NG2-glia, otherwise known as oligodendrocyte precursor cells (OPC). While, a generally agreed upon role of NG2+ cells is to generate oligodendrocytes, they are also thought to generate neurons and astrocytes (Dayer et al., 2005). Though, the latter remains controversial (Clarke et al., 2012). They are one of the largest proliferative cells in the adult cortex (Dawson et al., 2003). Nevertheless, not all NG2+ cells are proliferating at rest (Butt et al., 2005). Furthermore, a subset of them also appears to be involved in some aspects of information processing in partnership with neurons (Bergles et al., 2000; Lin and Bergles, 2002; Hamilton et al., 2009; Richardson et al., 2011). The varied functional roles of NG2-glia are not yet completely understood and await further studies (Peters, 2004; Richardson et al., 2011).

Nevertheless, it is clear that these cells are influenced by different manipulations, environmental and pharmacological, triggering its proliferation. Gliogenesis has been shown to be influenced by stress (Banasr et al., 2007; Czeh et al., 2007), exercise (Mandyam et al., 2007), growth factors (Elsayed et al., 2012), pharmacological and non-pharmacological modes of antidepressant treatment (Kodama et al., 2004; Wennström et al., 2006; Czeh et al., 2007). Furthermore, formation of new myelin is speculated to contribute to motor learning in humans (Richardson et al., 2011) as indicated by studies reporting changes in white matter structure following extensive piano practice (Bengtsson et al., 2005) or juggling (Scholz et al., 2009).

### Microglia

Microglial cells are the resident macrophage cells of the CNS. Unlike the other glia, they are ontogenetically related to the mononuclear phagocyte lineage. They act as a warden (CNS surveillance) and cleaner (macrophage). Microglial cells have a distinct morphology, small soma with fine and short processes. In 2010, a fate mapping study shed new light on the period of microglia migration to the brain. The study demonstrates that migration occurs during early embryonic development, challenging the idea that it enters the brain after birth (Ginhoux et al., 2010). Hence, this study indicates that migration of microglia coincides with neuronal development. This realization led to the identification of new roles of microglia in neuronal development and wiring in the healthy brain (Tremblay et al., 2010; Schafer et al., 2012). In addition, microglia are involved in CNS surveillance and maintenance. They have constantly motile cellular processes canvassing the extracellular space (Kettenmann et al., 2011; Wu et al., 2013) and are involved in synaptic pruning and refinement of neuronal circuits (Chu et al., 2010). Furthermore, microglial cells are involved in neuroinflammation. In response to a pathogen or injury, microglial cells once activated change in morphology. They proliferate, migrate to the site of injury (or infection) and they phagocytose damaged neurons, myelin and degenerating cells (Ginhoux et al., 2013). They are also involved in activating the immune system by releasing factors (such as cytokines and chemo-attractive factors) to promote neuronal protection and survival.

## Glia and Behavior

**Figure 1:**
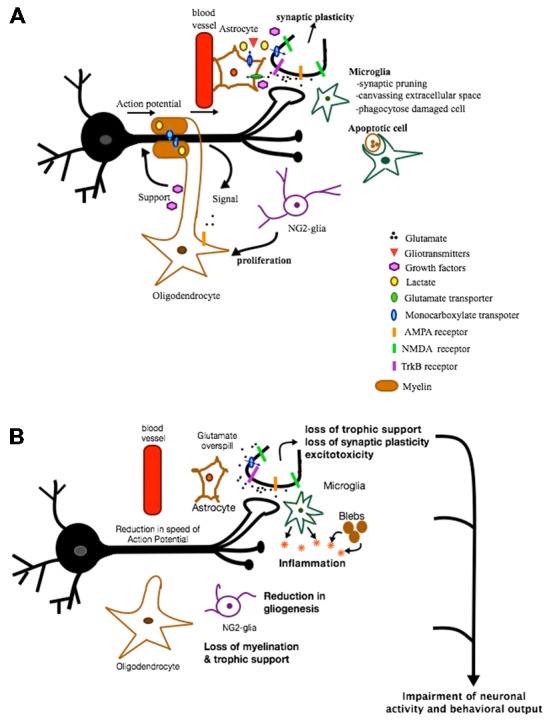
**Role of glia in health and disease.** This is a figure depicting some of the various roles glial cells can play under normal and pathological conditions. **(A)** In healthy conditions, astrocytes provide protective and metabolic support to neurons via the supply of trophic factors and metabolic products and via the reuptake of synaptic glutamate. In addition, some of the factors and gliotransmitters released are involved in inducing synaptic plasticity. Microglia are involved in synaptic pruning, canvassing extracellular space and phagocytosing apoptotic cells. NG2-glia act mainly as oligodendrocyte progenitor cells. When activated, they proliferate and differentiate into oligodendrocytes. Oligodendrocytes, on the other hand, support neurons metabolically, are neuroprotective, and provide a source of myelin necessary for proper propagation of action potential. These various functions of glia are all crucial for maintaining proper neuronal functioning and communication. **(B)** In pathological conditions, loss of different types of glia can result in loss of trophic support, loss of synaptic plasticity, excitotoxicity, inflammation, loss of myelination, etc. All of these effects can impair neuronal activity and function and ultimately behavior.

The diverse functions of glia discussed above clearly indicate that they are not just structural fillers. Rather, they play an integral part of functional communication in the brain, see Figure 1. Thus, it is not surprising to come across studies demonstrating the impact of glia on behavior. While neurons have long received attention as the main and ultimate drivers in inducing a behavioral output, glia are emerging as equally important influencers in certain behavioral aspects. One supportive study demonstrates cognitive and mood deficits following glial damage. Upon infusion of the gliotoxin L-alpha-aminoadipic acid (L-AAA) into the prefrontal cortex (PFC), anhedonia- and despair-like behaviors were manifested. Moreover, the gliotoxin triggered morphological changes in the neurons. Interestingly, inducing neuronal loss by infusion of the neurotoxin ibotenate into the PFC did not replicate these results. This indicates that glial dysfunction is an important player with capability of inducing depressive symptoms possibly by contributing to neuronal adaptive changes responsible in eliciting the expression of symptoms of depression (Banasr and Duman, 2008). Behavioral impairments were also found when targeting specific astrocytic activities. Following impairment of astrocytic vesicular release through genetic manipulation, gamma oscillations were found to be impacted and this was accompanied by a deficit in novel object recognition test indicating memory impairment (Lee et al., 2014). Another study examined the behavioral impact following impairment of glycogenolysis in the rodent hippocampus. Glycogenolysis is a process that occurs uniquely in astrocytes and involves break down of glycogen and lactate formation. Inhibiting this astrocytic function interfered with long-term memory formation. A similar behavioral output also occurred following manipulation of astrocytic export or intra-neuronal uptake of lactate. These findings suggest that manipulating one aspect of astrocyte function can have a strong impact on important physiological functions, such as long-term memory formation (Suzuki et al., 2011). Additional evidence comes from a recent study suggesting that glial cells have computational and cognitive enhancement abilities. The authors engrafted human glial progenitor cells into neonatal immune-deficient mice. At adulthood and upon maturation, these chimeric mice contain both mice and human astroglia. What was puzzling about these mice is that they exhibited enhanced learning and LTP when compared to mice allografted with murine glial progenitor cells (Han et al., 2013). This study alludes to the notion of astrocytic evolution geared towards the enhancement of our cognitive abilities. In sum, these studies are some of many emerging findings that strongly highlight the importance and impact of glia on modulating cognition and emotions. Since impairments in cognition and mood are features of mental illnesses, it makes sense to draw our attention to glia and the pathological findings reported in these cell types.

## Mental Illnesses

According to Center for Disease Control and Prevention (CDC), mental illnesses refer to disorders generally characterized by dysregulation of mood, thought, and/or behavior, as recognized by the Diagnostic and Statistical Manual DSM-IV. Unlike neurodegenerative disorders, mental illness is not characterized by significant loss of neurons but rather by a prominent glial pathology (Rajkowska, 2002b; Rajkowska and Miguel-Hidalgo, 2007).

## Major Depressive Disorder

### Clinical Studies

Major depressive disorder (MDD) is characterized by depressed mood, anhedonia and altered cognitive function. The experience of some of these symptoms can be disabling, interfering with one’s daily activities and function. In some cases, these disabling symptoms are recurrent; they may reappear several times in the lifetime of MDD patients.

MDD is a multifactorial brain disorder with both genetic and environmental components. Brain imaging and molecular pathology studies have identified alterations in key structures involved in the regulation of mood and cognitive functions. Functional neuroimaging studies measuring changes in glucose metabolism (Positron Emission Tomography), blood perfusion (functional Magnetic Resonance Imaging) and volumetric studies (Magnetic Resonance Imaging) show structural and functional alterations in the PFC, hippocampus, striatum and amygdala (Drevets, 1999, 2000, 2001; Zhu et al., 1999). More specifically, reports indicate a decrease in metabolism of dorsolateral PFC (dlPFC), subgenual anterior cingulate, and an increase in orbital cortex/ventrolateral PFC, posterior cingulate cortex (Drevets et al., 2002a) and amygdala (Drevets et al., 2002b). Though, normal and hyperfrontal normal activities have also been reported (Mayberg, 2003; Fales et al., 2008) indicating some inconsistencies. The decrease in some of the cortical activity in depressed patients is restored following antidepressant treatment (Mayberg et al., 2000; Liotti and Mayberg, 2001). Amygdala activity is generally under negative control by PFC; the general decrease in PFC function and increased amygdala activity point to a disrupted circuitry. Indeed, studies have reported decreased prefrontal-amygdala functional connectivity (Matthews et al., 2008; Almeida et al., 2009). This is consistent with impaired cognitive regulation of negative emotions, a commonly experienced symptom by depressed subjects. The disruption of this circuitry is further substantiated by anatomical studies indicating cellular and myelination changes in many of these brain regions (Zhu et al., 1999; Manji et al., 2001).

One of the earliest reports of glia pathology dates back to 1998, when a preliminary histopathological assessment of subgenual part of Brodmann’s are (BA24) indicated reduction in gray matter volume and diminution in glial density with no changes in neuronal density in familial forms of MDD and bipolar disorder (BPD; Ongur et al., 1998). Further cellular characterization was conducted indicating changes in different glial cell types (Rajkowska et al., 1999). Glia pathology in MDD has become well documented. Table 1 lists some of these quantitative studies. Although there are numerous reports substantiating glial reductions in different limbic brain regions, there are some studies indicating otherwise. For a more detailed review, please refer to Sanacora and Banasr (2013).

**Table 1:** **Summary of the findings of glial cell reductions within the brain of depressed**.

Cortical region	Glial density	Neuronal size	Reference
DLPFC	Decrease in glia	20%	Cotter et al. (2002)
Supra and infragranular layers of	Dec. glia	5–7%	Rajkowska et al. (1999)
DLPFC and OrbitoFrontal Cortex (OFC)
Subgenual Anterior Cingulate changes in neuronal size/shape	Dec. in Glia (Area 24) across all layers	Selective decrease in layer Vb and	Gittins and Harrison (2011)
Anterior cingulate cortex	Dec. in Glia density layer 6 (22%)	23% layer 6	Cotter et al. (2001)
Hippocampus (CA1 region)	Dec. in astrocytes	Not reported	Gos et al. (2013)
Amygdala	Dec. in glia and oligodendrocytes	Not reported	Bowley et al. (2002) and Hamidi et al. (2004)

Studies on specific markers for oligodendrocytes have shown a decrease in frontal cortex (Honer et al., 1999), in middle temporal gyrus (Aston et al., 2005), in deep white matter of the dlPFC (Regenold et al., 2007) and in white matter volume of genual and splenial portions of corpus callosum (Brambilla et al., 2004). The greatest changes were observed in layers III, V, and VI (Rajkowska et al., 1999; Cotter et al., 2001, 2002; Rajkowska, 2002b; Uranova et al., 2004; Banasr et al., 2007). Given the presence of a large component of myelinated fibers in these deeper layers, these findings support the hypothesis that myelinating oligodendrocyte’s function is reduced in MDD. Reductions in limbic regions such as the amygdala (Hamidi et al., 2004) were also observed. Furthermore, white matter alterations in the anterior cingulate, dlPFC and central white matter regions were observed with Diffusion Tensor imaging and results suggest that disconnections of cortical and subcortical regions occur with depression (Bae et al., 2006).

In addition, changes in expression of astrocytic markers critical to the function and regulatory mechanisms of astrocytes have been reported in dlPFC, anterior cingulate cortex, orbitofrontal cortex and locus coeruleus. A number of postmortem brain studies of depressed subjects have consistently shown reductions in the expression of GFAP (Miguel-Hidalgo et al., 2000), AQP4 (Rajkowska et al., 2013), connexins (Miguel-Hidalgo et al., 2014), S100B (Gos et al., 2013), glutamate transporters and glutamine synthase expression (Choudary et al., 2005; Medina et al., 2013) and TrkB.1, an isoform specifically expressed in astrocytes (Ernst et al., 2009). One study identified a significant reduction (by 50%) in the coverage of blood vessels by astrocytic end feet in the gray matter of the orbitofrontal cortex (Rajkowska et al., 2013). Being an active participant in the neuro-vascular unit and a metabolic coupler of neuronal activity with blood glucose uptake, this suggests that there is a strong impairment in this particular metabolic astrocytic activity. Hence, it is not surprising that these cellular changes, particularly the coverage of blood vessels are accompanied with metabolic changes in this particular brain region when examined in anxious depressed subjects (Townsend et al., 2010). To identify the etiological mechanism of astrocytic pathology in depression, changes in DNA methylation patterns were reported in astrocytes that were specifically altered in the brain of depressed subjects (Nagy et al., 2015). Reductions of astrocyte related marker GFAP was however, not observed in the older MDD subjects (46-86 years of age; Miguel-Hidalgo et al., 2000). The lack of effect in the older MDD population is thought to be due to age-related astrocyte reactivity.

With regards to microglia, clinical evidence implicating microglial dysregulation in MDD is limited. While no studies to date have reported a loss of microglia, one study found significant microgliosis in dlPFC, anterior cingulate cortex and mediodorsal thalamus of suicidal subjects (Steiner et al., 2008). Furthermore, quinolinic acid and pro-inflammatory cytokines, whose main source of production and release is microglia are elevated in areas within anterior cingulate cortex and in the cerebrospinal fluid respectively in a subgroup of depressed subjects (Howren et al., 2009; Steiner et al., 2011). Readers are referred to a recent review that thoroughly discusses the findings and the potential role of activated microglia in the pathophysiology of MDD and other neuropsychiatric disorders (Beumer et al., 2012).

These cellular alterations are thought to be the underlying mechanism for the structural changes and volumetric reductions observed in specific brain regions of MDD subjects. Dysfunction of glial cells, glial loss and/or reduced gliogenesis (Rajkowska et al., 1999; Cotter et al., 2001, 2002), are possible mechanisms that could lead to the reported neuronal atrophy and impairment in neuronal functioning and output.

### Preclinical Studies of Depression

The importance of glia in mood modulation has been highlighted recently in animal models of depression. An increase in cell proliferation in the PFC of adult rats at baseline levels occurs after 3 weeks of antidepressant treatment (Kodama et al., 2004). Chronic stress and chronic corticosterone administration result in at least 30% decrease of cell proliferation in the medial PFC and cerebral cortex of adult rats (Alonso, 2000; Banasr et al., 2007; Czeh et al., 2007) and mice (Elsayed et al., 2012). Some of these changes are reversed by chronic fluoxetine treatment (Banasr et al., 2007; Czeh et al., 2007). Furthermore, the nature of the proliferative cells decreased by stress in the PFC was identified as NG2+ cells and endothelial cells (RECA-1+ marker; Banasr et al., 2007; Elsayed et al., 2012). This decrease in cell proliferation following chronic stress was accompanied by a reduction in the number of newly generated oligodendrocytes (RIP+; Banasr et al., 2007; Elsayed et al., 2012). These studies suggest that reductions in cortical cell proliferation could contribute to the glial alterations observed in depressed patients and that antidepressants might act in part by blocking or reversing these effects. In addition, rodent chronic stress and maternal deprivation models of depression also resulted in reduced astrocyte numbers and density in the hippocampus (Leventopoulos et al., 2007; Araya-Callis et al., 2012) and PFC (Banasr et al., 2010).

Factors that may contribute to a loss of glia include the alterations in glucocorticoid secretion and glutamatergic transmission evident during depression. Elevated glucocorticoid concentration, as a result of repeated stress, can decrease the proliferation of OPC (Alonso, 2000) and astrocyte density (Nichols et al., 1990). Furthermore, the early loss of glia in the initial stages of depression may lead to a reduction in glutamate clearance from the synaptic cleft. As a result, extracellular glutamate levels rise and can contribute to further glial damage (Rajkowska and Miguel-Hidalgo, 2007). Indeed, astrocytes, OPC and mature oligodendrocytes express glutamate receptors making them responsive to glutamate signaling and susceptible to excitotoxic damage from excess glutamate (Matute et al., 1997; McDonald et al., 1998; De Biase et al., 2011; Vielkind et al., 1990).

Astrocytes are functionally diverse and can exert a significant impact on cognitive and emotion-related behaviors. One study has shown that by interfering with astrocytic gliotransmission and vesicular ATP release, animals exhibit depressive-like symptoms (Cao et al., 2013). Furthermore, mice with knock-out of the gene encoding for Aquaporin-4, a protein predominantly expressed in astrocytes, show loss of astrocytes and exacerbated depressive-like behaviors when subjected to chronic corticosterone treatment (Kong et al., 2014). Together, these studies suggest that astrocytic pathology is implicated in the pathogenesis of depression.

Preclinical studies also suggest the involvement of microglial activity in the expression of depressive symptoms. Upon challenging the immune system and activating microglia, rodents express depressive-like symptoms that can be reversed with chronic antidepressant treatment (Yirmiya, 1996; Yirmiya et al., 2001). Furthermore, microglial activity is sensitive to antidepressant treatment and chronic stress (Hashioka et al., 2007; Goshen and Yirmiya, 2009). Chronic stress increases microglia activation in the rat PFC, an effect reversed by minocycline, an antibiotic that blocks microglial function (Hinwood et al., 2012) and exerts antidepressant effects (Arakawa et al., 2012). Further evidence implicating this cell type comes from studies on mice deficient in the fractalkine receptor CX3CR1 which is exclusively expressed by microglia; these mice exhibit depressive-like behaviors following activation of microglia by lipopolysaccharide treatment (Corona et al., 2010).

Interestingly, *in vitro* studies of pure glial cell cultures indicate a direct action of antidepressants on glia, in addition to their classical effects on monoaminergic neurons. Studies show that antidepressants can enhance astrocytic metabolism (Zhang et al., 1993; Kong et al., 2002; Allaman et al., 2011), increase growth factors expression (Allaman et al., 2011; Kajitani et al., 2012), and reduce the production of inflammatory cytokines (Obuchowicz et al., 2014). These *in vitro* studies point to a variety of glial-mediated mechanisms that could underlie therapeutic effects of antidepressants. The metabolic effects of antidepressants observed in culture could also explain the clinical findings indicating a recovery in glucose metabolism in affected brain regions following antidepressant treatment (Mayberg et al., 2000; Drevets et al., 2002a).

## Bipolar Disorder

BPD, also known as manic-depressive illness is another classification of mood disorders. It is characterized by fluctuating moods between depression and mania, and both phases can occur with psychotic features. Moreover, unlike MDD it has a significant genetic component.

Structural and functional neuroimaging studies have indicated volumetric changes in cortico-limbic brain regions (Ongur et al., 1998; Rajkowska, 2002a) and compromised white matter integrity in BPD (Hercher et al., 2014). In addition to alterations in density/size of specific types of cortical neurons, glia loss has been reported to occur in postmortem brain studies of BPD subjects. Loss of glia is up to 40% in mPFC (area 24) as indicated by a study of a small cohort of familial BPD, and was consistent with the neuroimaging findings (Drevets et al., 1997; Ongur et al., 1998). Furthermore, unlike MDD, these glial reductions are lamina-specific within the dlPFC (Rajkowska et al., 2001) and are accompanied by glial hypertrophy (Rajkowska et al., 2001). Glial reductions also extend to subcortical regions such as the amygdala (Bowley et al., 2002) but do not appear to be widespread (i.e., there is no change in glia density in the supracallosal part of anterior cingulate cortex; Cotter et al., 2001). Hence, these results suggest reductions in glia density within regions of the cortico-limbic structures, particularly within the ventrally located regions and more subtle changes within dlPFC.

Studies delineating the specific types of glia involved suggest a severe loss of oligodendrocytes and myelin with some mixed results concerning astrocytes. In a microarray and qPCR study, markers for oligodendrocytes which include myelination related genes such as PLP1, MAG, CLDN11, MBP, MOG, GALC and Transferrin were decreased in the PFC of BPD subjects (Tkachev et al., 2003). Abnormalities in satellite oligodendrocytes were also evidenced by electron microscopic analysis of PFC in BPD (Uranova et al., 2001; Vostrikov et al., 2007; Drevets et al., 2008) suggesting oligodendrocyte dysfunction. On the other hand, decreased Glycogen Synthase Kinase 3 (GSK3) activity, as a result of lithium treatment or variation in the promoter of GSK3 gene seen in some patients, is associated with enhancement in white matter integrity and improvement in clinical feature of BDP (Benedetti et al., 2013). These results suggest the involvement of oligodendrocyte/myelin integrity in the development and treatment of BPD symptoms.

There are some mixed results when it comes to astrocytic reductions. One study reported a decrease in GFAP immunostaining across all layers of the orbitofrontal cortex while another study found no changes in the subgenual cingulate cortex (Toro et al., 2006; Williams et al., 2013) despite a decrease in GFAP mRNA expression in the dorsal portion (Webster et al., 2005). Furthermore, the expression of another astrocytic marker, glutamine synthase, was not changed in the dorsolateral and in the orbitofrontal cortex (Toro et al., 2006).

### Preclinical Studies of Bipolar Disorder

To date, there is no established animal model of BPD that exhibit mania-like symptoms, particularly alternating episodes of mania and depression-like behaviors. Hence, the field has relied mostly on genetic studies and/or examining the effects of two commonly used and oldest therapeutic drugs, Lithium and Valproate. One study found that chronic lithium treatment decreases NG2 cell proliferation within the hippocampus (Orre et al., 2009), and reduces glycogen synthesis in astrocytes (Souza Ade et al., 2010). Furthermore, lithium within therapeutic concentrations was found to inhibit GSK3 activity (Bain et al., 2007). One of the numerous effects of GSK3 is to regulate oligodendrocyte differentiation and myelination (Azim and Butt, 2011). As such, this can represent a mechanism by which lithium can exert therapeutic effects via possible regulation of oligodendrocyte differentiation and function. Additional targets include neuregulin and its receptor, erbB4 that are genetically linked to BPD and are implicated in oligodendrocyte development (Roy et al., 2007).

These findings suggest that treatment of BPD could be exerted via modulation of different pathways that regulate the activities and functioning of different glial cell types. Nevertheless, more preclinical studies would need to be carried out to further establish this link.

## Anxiety Disorders

Anxiety disorders have a high degree of comorbidity with MDD. While anxiety is a natural response to a life threatening situation, it becomes a disorder when it disrupts one’s daily activities. There are six subtypes of anxiety disorders defined by specific symptoms: generalized anxiety disorder (GAD), panic disorder (PD), obsessive-compulsive disorder (OCD), post-traumatic stress disorder (PTSD) and agora- or socio-phobias. All have a common feature which is a lack of correct processing of fear stimuli.

Imaging and magnetic resonance studies report brain structural alterations, (Li et al., 2014) and glial metabolite changes in different subtypes of anxiety disorders (Seedat et al., 2005; Kitamura et al., 2006). Furthermore, disruption in myelin integrity and structure has been observed particularly in OCD subjects within fronto-striato-thalamo cortical circuit. These structural changes were associated with a functional polymorphism in the myelin oligodendrocyte glycoprotein (MOG; Atmaca et al., 2010). White matter contains fiber tracts, surrounded by myelin sheaths and fibrous astrocytes. These studies indicate that disturbed myelination/oligodendrocyte function and/or astrocytic function are implicated in anxiety disorders. Additional studies showed an association between the polymorphisms in oligodendrocyte lineage transcription factor OLIG2 and OCD, further supporting white matter and oligodendrocyte abnormalities in this disorder (Stewart et al., 2007).

In addition, Riluzole, a drug used in the management of ALS has been shown to exert beneficial effects in patients with OCD (Coric et al., 2003) and GAD (Mathew et al., 2005; Pittenger et al., 2008). Riluzole can act on astrocytes and enhance astrocytic uptake of extracellular glutamate in addition to other effects. Given the numerous mechanisms it can exert, it is remains to be determined whether its therapeutic effect is via enhancement of astrocytic uptake of glutamate *per se*.

### Preclinical Studies

While the most consistent pathological findings in postmortem studies were disruptions in myelination, few preclinical studies have been carried out to examine a causal link between oligodendrocytes and anxiety. Rodents exposed to cuprizone, a demyelinating drug, exhibit anxiety related behavioral responses (Serra-De-Oliveira et al., 2015) thus providing a potential link between impairment in myelin/oligodendrocyte functioning and anxiety.

The association between astrocyte activation and different behavioral forms of anxiety has been explored to some extent. In one study, the metabolic and metabolite effects of antidepressant/antipanic drug phenelzine in rat cortex was examined using H1[13C]magnetic resonance spectroscopy. The rate of glutamate-glutamine cycling flux between neurons and glia was significantly reduced following treatment (Yang and Shen, 2005). A transcriptome analysis performed in the amygdala of rats exposed to fear learning, a behavioral model of Posttraumatic Stress Disorder (PTSD), showed induction of 84-astrocyte-enriched genes following shock exposure (Ponomarev et al., 2010). This indicates a possible involvement of astrocytes within the amygdala in stress-associated behavioral response. Expression of FGF2, predominantly expressed in astrocytes, is decreased in rats selectively bred for high anxiety. Treatment with FGF2, on the other hand, has anxiolytic effects. In addition, this treatment regimen results in increased hippocampal neurogenesis and gliogenesis pointing to the possible involvement of these cellular processes in anxiety modulation (Perez et al., 2009). In another model of PTSD that involves single prolonged stress (SPS), FGF2 administration was shown to inhibit SPS-induced hyperarousal and anxiety behavior, symptoms resembling PTSD. This was accompanied by a specific upregulation of GFAP expression in the hippocampus indicating that the anxiolytic effects of FGF2 could involve astrocyte-based mechanisms (Xia et al., 2013). Furthermore, riluzole administration in the medial PFC has been shown to block anxiety-like behavior indicating the possible involvement of astrocytic function in modulating anxiety via enhanced uptake of extracellular glutamate (Ohashi et al., 2015).

With regards to microglial involvement, knock-out mice for the Hoxb8 gene, a homeobox developmental patterning gene expressed prominently in the macrophage-lineage of hematopoietic cells and expressed by a subset of microglia, exhibit OCD-like behaviors which can be normalized following repopulation of the brain with wild-type microglia (Chen et al., 2010).

## Schizophrenia

Schizophrenia is a chronic and disabling neurodevelopmental disorder with polygenic and environmental factors playing a role. It is characterized by positive symptoms such as delusions, hallucinations, disordered thoughts, and negative symptoms such as deficits of normal emotional responses and thought processes. While the positive symptoms are in general better controlled with antipsychotics, negative symptoms are not.

Schizophrenia is regarded as a syndrome of inter- and intra-hemispheric disconnectivity particularly that of reduced cortical connectivity, for which the underlying biological and genetic cause remains unclear. While cell biology studies have predominantly focused on neurons, multiple lines of evidence from neuroimaging, postmortem brains and genome-wide associations implicate oligodendrocyte abnormalities and compromised white matter/myelin integrity (Dwork et al., 2007; Bernstein et al., 2015). Genetic and protein expression studies in schizophrenia indicate abnormalities in Myelin associated markers (Flynn et al., 2003; Iwamoto et al., 2005) within the cortex (Aston et al., 2004; Aberg et al., 2006; Tkachev et al., 2007) and within subcortical brain regions (Dracheva et al., 2006; Barley et al., 2009) with the most profoundly affected brain regions being the hippocampal formation, cingulate and temporal cortices (Katsel et al., 2005a,b). One mechanism contributing to this oligodendrocyte/myelin abnormality could be linked to disrupted-in-schizophrenia-1 (DISC1) gene. DISC-1 disruption as a result of chromosomal translocation reduces expression of Neuregulin and its receptor ErbB3. These are some of several altered genes associated with the development of Schizophrenia (Millar et al., 2000; Blackwood et al., 2001; Hakak et al., 2001; Corfas et al., 2004; Silberberg et al., 2006). Being expressed by different cell types including oligodendrocytes (Deadwyler et al., 2000; Osbun et al., 2011), these proteins exert a variety of functions including regulating oligodendrocyte development, differentiation and CNS myelination (Vartanian et al., 1999; Taveggia et al., 2005; Chen et al., 2006; Hattori et al., 2014). Furthermore, postmortem histology studies indicate reductions in glial cells in anterior cingulate cortex (Stark et al., 2004) including decreases in oligodendrocyte density (Uranova et al., 2004) in hippocampus (Schmitt et al., 2009), in the perineuronal PFC (Vostrikov et al., 2007) as well as layer specific oligodendrocyte reductions in the dlPFC (Hof et al., 2003). This is accompanied with volumetric reductions, abnormalities in adulthood myelination in the frontal lobes, association areas (Bartzokis et al., 2003) and temporal lobes (Chambers and Perrone-Bizzozero, 2004) and in white matter fiber tracts interconnecting brain regions, particularly the frontal and temporal lobes (Breier et al., 1992; Paillere-Martinot et al., 2001). It was hypothesized that the abnormalities in oligodendrocyte/myelin are possibly due to alterations in proliferation and differentiation of oligodendrocyte progenitor cells, NG2. Indeed, a microarray study carried out on the brains of schizophrenic patients revealed changes in gene expression associated with the regulation of G1/S phase transition and oligodendrocyte differentiation (Katsel et al., 2008). In support of a cell cycle impairment, variation in OLIG2, a gene strongly implicated in the control of oligodendrocyte development, was identified as a susceptibility gene in schizophrenia (reviewed in Georgieva et al., 2006). In addition, the reduction of perineuronal non-myelinating oligodendrocytes suggests impairments in oligodendrocyte functions that are beyond myelination. Together, these studies indicate that inadequate myelination or myelin function, abnormalities in oligodendrocyte development, density and functions could contribute to the pathophysiology and expression of schizophrenia symptoms.

Findings of abnormalities of astrocytes were less consistent and not as well surveyed. Examination of the astrocytic GFAP marker yielded differential results when examining it in various affected brain regions. Studies examining GFAP expression in cortical gray matter have identified no changes (Falkai et al., 1999; Katsel et al., 2011a), decreased expression (Johnston-Wilson et al., 2000; Steffek et al., 2008) or increased expression (Pennington et al., 2008; Feresten et al., 2013). Furthermore, some studies reported specific changes restricted to subgroups of schizophrenic subjects (Arnold et al., 1996). In sum, studies using GFAP as an astrocyte marker have yielded inconsistent results. Since GFAP may not represent a direct link to astrocyte density, other astrocytic markers were examined. The expressions of a few selected markers were found altered implying possible changes in specific astrocytic functions and/or astrocytic subsets (Owen et al., 1987; Katsel et al., 2011a; Feresten et al., 2013). These changes in astrocytic markers were glutamate-related, an observation consistent with the view that schizophrenia is associated with a hypofunction of glutamatergic transmission. Supporting this, the expression of astrocytic glutamate transporter was found increased in the PFC of schizophrenic subjects (Matute et al., 2005; Lauriat et al., 2006) and normalized following antipsychotic treatment (Matute et al., 2005), while that of glutamine synthase was decreased in the deep layers of the anterior cingulate (Steffek et al., 2008).

Microglial cells are also altered in the brain of schizophrenic patients. Cytology and imaging studies report increased number of activated microglia in the frontal and temporal lobes of schizophrenic patients (Bayer et al., 1999; Radewicz et al., 2000; Wierzba-Bobrowicz et al., 2005; Van Berckel et al., 2008). Activation of microglia can result in the release of proinflammatory cytokines and free radicals that can lead to abnormalities in white matter and neurons and thus in the expression of schizophrenia symptoms. Interestingly, minocycline, an inhibitor of microglial activation was found to have therapeutic benefit when used as an adjunctive treatment (Miyaoka et al., 2007; Levkovitz et al., 2010; Chaudhry et al., 2012). These findings indicate overactive microglia may play an important role in the pathophysiology of schizophrenia.

### Preclinical Studies

Animal models of schizophrenia face serious and vexing challenges given the complexity and difficulty to recapitulate the symptoms of schizophrenia. Nevertheless, preclinical studies have shed light and provided key insights into the involvement of the different glial cells in the pathophysiology of schizophrenia. For instance, demyelination and downregulation of oligodendrocyte-associated genes in PFC was shown to induce behavioral deficits associated with schizophrenia (a deficit in the ability to shift between perceptual dimensions in the attentional set-shifting task; Gregg et al., 2009). Mice with selective ErbB3 receptor deletion in oligodendrocytes exhibit deficits in social interaction and working memory (Makinodan et al., 2012). Transgenic mice expressing mutant human DISC1 specifically in the forebrain also show behavioral deficits similar to schizophrenia. In addition, these mice were found to exhibit premature oligodendrocyte differentiation and increased proliferation of their progenitors (Katsel et al., 2011b). These studies indicate that oligodendrocyte functional impairment via ErbB3 signaling or alterations in DISC1 function can contribute to schizophrenia pathogenesis and symptoms. Furthermore, transgenic mice with a deficiency of DISC1 expression in astrocytes have impaired D-serine production which in turn can affect NMDAR activity. These mice also display schizophrenia like-behaviors (prepupulse inhibition in the acoustic startle tests) consistent with hypofunction of glutamatergic transmission via NMDA receptors (Ma et al., 2013). Furthermore, pharmacological upregulation of the astrocytic glutamate transporter Glt-1 expression result in impairment of information processing, mimicking what occurs in schizophrenia (Bellesi et al., 2009). In an animal model of schizophrenia based on maternal infection during pregnancy, microglia activation in brain regions involved in the pathogenesis of schizophrenia i.e., hippocampus and striatum was observed (Juckel et al., 2011; Mattei et al., 2014). The behavioral deficits triggered in this animal model of schizophrenia were rescued following treatment with minocycline (Mattei et al., 2014). Lastly, antipsychotics have been shown to modulate microglial activity promoting anti-inflammatory effects (Labuzek et al., 2005; Kato et al., 2007). In sum, these findings support the involvement of microglia, oligodendrocyte and astrocytes in the pathophysiology and treatment of schizophrenia.

## Attention Deficit Hyperactivity Disorder

Attention deficit hyperactivity disorder (ADHD) is a highly heritable disorder characterized by a heterogeneous set of symptoms that include problems in attention, impulsivity and hyperactivity. Compelling lines of evidence indicate that symptoms of ADHD are associated with hypofunctionality of catecholaminergic pathways projecting to prefrontal cortical areas (Biederman and Spencer, 2000; Semrud-Clikeman et al., 2000; Todd and Botteron, 2001). For instance, unmedicated ADHD subjects exhibit increased dopamine transporter concentrations (Dougherty et al., 1999; Krause et al., 2000) that are normalized following treatment (Krause et al., 2000). It is well known that catecholamines can trigger glycogenolysis in astrocytes followed by lactate release (Magistretti, 1988; Sorg and Magistretti, 1991; Magistretti et al., 1993). It is hypothesized as such that catecholamine hypofunction could result in diminished activation of astrocytic energy metabolism and supply to prefrontal cortical neurons (Semrud-Clikeman et al., 2000; Russell et al., 2006; Killeen et al., 2013). In turn, rapid synchronized neuronal firing can be impaired, which might result in disturbances in neurotransmission (Todd and Botteron, 2001). This is supported by some imaging studies indicating changes in cerebral blood flow and glucose metabolism in ADHD subjects (Zametkin et al., 1990, 1993; Ernst et al., 1994; Gustafsson et al., 2000; Hart et al., 2012) though these findings were not always reproducible in other cohorts (Ernst et al., 1997). The inconsistencies in these imaging studies might be related to the phenotyping heterogeneity of the disease. In addition, ADHD individuals exhibit altered myelination and disrupted network connectivity (Fair et al., 2010; Nagel et al., 2011). Since Lactate is involved in myelin production (Rinholm et al., 2011), it is conceivable that this deficit in energy supply in the form of Lactate could also interfere with myelin production and hence neuronal transmission.

### Preclinical Studies

Spontaneously hypertensive rats (SHR) display hyperactivity, impulsivity and poor performance in tasks that require sustained attention. Thus, they represent a model of ADHD. These rats show reductions in proteins involved in energy metabolism and myelination (Dimatelis et al., 2015). Given that glia particularly astrocytes are key players in brain energy metabolism, this finding further support a role of astrocytic energy metabolism deficit in ADHD genesis. Further evidence supporting glial contribution to the pathophysiology of this disorder is provided by a study showing that mutant mice with a disrupted SynCam1 specifically in astrocytes result in behavioral deficits related to ADHD symptoms (Sandau et al., 2012).

## Substance Use Disorders

Substance use disorders (SUD) is a chronic brain disorder with profound effects on our society. Addicted individuals untiringly seek substance of abuse despite the negative consequences associated with it. Apart from the huge economic burden it carries, SUD have devastating consequences on society and quality of life. Vulnerability to addiction is influenced by genetics, environmental factors and developmental stages (Volkow et al., 2011). Chronic drug abuse impairs many aspects of behavior necessary for proper functioning in social environment. For example, alcohol dependance leads to impairments in executive function and episodic memory (Bernardin et al., 2014). These impairments are seen as a result of structural and functional changes in limbic circuits and frontal brain regions. Indeed, imaging studies indicate volumetric changes in the frontal lobe in cocaine-, alcohol- and heroin-dependent subjects (Goldstein and Volkow, 2002). While neuronal dysfunction particularly dopaminergic, glutamatergic and opioidergic transmissions are the underlying pathophysiological mechanisms, pathological changes in glial cells are also observed (Miguel-Hidalgo, 2009).

Alcohol dependent subjects exhibit reductions in glial densities in dlPFC (Miguel-Hidalgo et al., 2002), orbitofrontal cortex (Miguel-Hidalgo et al., 2006) and hippocampus (Korbo, 1999). Glial loss includes astrocytes and oligodendrocytes. While there are also some neuronal losses, the deficit is not as widespread. Unlike glia, there is no loss of neurons in the hippocampus (Korbo, 1999) and it is limited to specific cortical layers of the orbitofrontal cortex (Miguel-Hidalgo et al., 2006). Several findings based on examination of a number of glial markers substantiate glial pathology in alcoholic subjects. For example, connexin 43, an astrocytic gap junction, is significantly reduced in the orbitofrontal cortex of alcoholics indicating impairment in astrocytic communication (Miguel-Hidalgo et al., 2014). Furthermore, a mutation in a glutamate transporter specifically expressed in astrocytes, GLT-1, was found to increase vulnerability to alcohol dependance (Sander et al., 2000). In addition to astrocytic pathologies, postmortem studies of the brains of alcohol dependent subjects indicate increased expression of microglial markers in specific brain regions (He and Crews, 2008) and altered oligodendrocyte/myelin gene expression indicating white matter dysfunction (Lewohl et al., 2000; Pfefferbaum et al., 2000; Mayfield et al., 2002; Liu et al., 2004). Some of these changes in oligodendrocyte markers and the expression of several myelination related genes were also observed in cocaine abusers (Albertson et al., 2004; Bannon et al., 2005).

### Preclinical Studies

Preclinical studies helped dissect the role of these pathologies play in addiction. Studies demonstrating impairment in astrocyte density/function particularly those pertaining to glutamate homeostasis are of particular interest. Chronic exposure to both cocaine and nicotine in rodents resulted in reduced expression of a catalytic subunit of cysteine glutamate antiporter expressed predominantly in glia (Kalivas et al., 2003; Kalivas, 2009). Exposure to other forms of substances of abuse (i.e., alcohol, heroin, etc.) was also shown to result in reduced expression levels of GLT-1 in the nucleus accumbens (Kalivas, 2009; Sari and Sreemantula, 2012; Gipson et al., 2013). While gene expression levels of GLAST, another glutamate transporter subtype, were found increased in the frontal cortex in alcohol-dependent rodents (Rimondini et al., 2002), GLT-1 mediated functions seem to be disrupted in this brain region (Mulholland et al., 2009). The reduction in glutamate re-uptake particularly in nucleus accumbens seems to be a consistent maladaptive response to these different drugs of abuse. This pathology may result in potentiation of glutamatergic transmission and in activation of non-synaptic glutamatergic compartment which is associated with drug seeking behavior (Kalivas, 2009; Scofield and Kalivas, 2014). Additional aspects of astrocytic functions seem to also be implicated. Alcohol preferring rats show increased GFAP-immunoreactive cells following few weeks of exposure (Miguel-Hidalgo, 2005) while longer duration resulted in reduction in perineuronal glial cell densities (Khokhrina et al., 1991). Furthermore, alcohol self-administration is increased following infusion of the gliotoxin L-AAA or astrocytic gap junction blockers into the prelimbic cortex (Miguel-Hidalgo et al., 2009). In addition to the astrocytic pathologies and consistent with clinical findings, chronic exposure to alcohol also results in a decrease in oligodendrocyte/myelin gene expression (Okamoto et al., 2006). Interestingly, the identification of a glial modulator, ibudilast was shown to exert therapeutic effects in rodent models of addiction (Snider et al., 2013; Bell et al., 2015).

Taken together, these studies suggest that alcohol and additional substances of abuse can have profound effects on glial density and function in relevant brain regions. Furthermore, interfering with glial density and/or function seems to affect vulnerability for addiction. Thus targeting specific functions of glia could represent a new therapeutic avenue.

## Alexander Disease

Alexander disease is a rare and fatal disease of the CNS, predominantly affecting infants and children. Affected patients suffer from cognitive and motor impairments in the form of mental retardation, seizures, megaloencephaly and progressive deterioration (Prust et al., 2011; Verkhratsky et al., 2014). The pathology is a glial one associated with sporadic mutations in the non-conservative coding region of GFAP (Brenner et al., 2001; Rodriguez et al., 2001). These mutations are thought to result in cytotoxicity. Indeed, histological analysis has indicated cytoplasmic inclusions in astrocytes that contain the intermediate filament GFAP, otherwise referred as Rosenthal fibers. It is thought that these fibers, represent the hallmark of this disease (Sawaishi, 2009). Furthermore, variable degrees of cerebral white matter degeneration, referred as leukodystrophies, have been observed prominently in the frontal lobes (Messing et al., 2012) and in close apposition to Rosenthal fibers. Since astrocytes can release factors involved in myelination (Ishibashi et al., 2006; Sawaishi, 2009), it is thus speculated that white matter abnormalities are a consequence to astrocytic pathology.

### Preclinical Studies

To further cement the involvement of this astrocytic genetic defect in Alexander disease pathology, mouse models of Alexander Disease overexpressing human GFAP mutation were generated. A similar astrocytic pathology with inclusions of Rosenthal fibers was observed (Eng et al., 1998). In addition, decreased glutamate transporter levels that were also reported in human subjects (Tian et al., 2010) were demonstrated along with cognitive impairments (Hagemann et al., 2013).

In sum, these findings present Alexander disease as a primary astrocytic genetic disorder. Impairment in astrocytic function via decreased glutamate uptake and/or release of factors involved in myelin formation, can trigger the pathogenesis of neuronal and oligodendrocyte injury/death and ultimately manifesting symptoms of Alexander disease.

## Conclusion

Converging lines of evidence from clinical and preclinical studies suggest that different types of glial cells can play a substantial role in the pathology of mental illnesses. Furthermore, there appears to be an overlap in glial pathologies in some of the mental illnesses pointing to the multi-functional impact of these cells in the expression of diverse symptoms. For example, reports of reductions in glial density within the dorsolateral PFC are indicated in subjects diagnosed with depression (Rajkowska et al., 1999) and alcoholism (Miguel-Hidalgo et al., 2002). A direct cause effect is further demonstrated in preclinical studies whereby injection of a gliotoxin into the PFC results in behavioral effects associated with depression (Banasr and Duman, 2008) and alcohol preference (Miguel-Hidalgo et al., 2009). These overwhelming findings implicating glial cells in the pathophysiology of mental illnesses should alter our perception of mental illnesses. It should also promote interest towards targeting glial cells as a new avenue of treatment. Table 2 below is a summary of the glial pathological findings reported among the different types of mental illnesses and a list of compounds with therapeutic benefits that target different types of glial cells, in hope to shed light on these cast-aside cells that seem to hold more potential than we think.

**Table 2:** **Summary of the therapeutic drugs that target glial cells**.

Neuropsychiatric disorder	Glial cells affected	Therapeutics targeting glial cells	Glial cells responsive to therapeutics
Major depressive disorder	Astrocytes	Riluzole (Yoshizumi et al., 2012)	-Astrocytes
	Microglia	Fluoxetine, Paroxetine (Allaman et al., 2011)	-Oligodendrocyte
	Oligodendrocytes	Ceftriaxone (Li et al., 2012)	progenitors
		Minocycline (Hinwood et al., 2012)	-Microglia
Bipolar disorder	Oligodendrocytes	Lithium (Orre et al., 2009; Souza Ade et al., 2010)	-Astrocytes
	Astrocytes		-Oligodendrocyte
	Microglia		progenitors
Anxiety disorders	Oligodendrocytes	Riluzole (Pittenger et al., 2008)	-Astrocytes
	Astrocytes	Benzodiazepines (Patte et al., 1999)
Schizophrenia	Astrocytes	Minocycline (Chaudhry et al., 2012)	-Microglia
	Microglia	Risperidone (Kato et al., 2007)
	Oligodendrocytes	Chlorpromazine and loxapine (Labuzek et al., 2005)
Substance use disorders	Astrocytes	Ibudilast, AV1013, minocycline (Snider et al., 2013)	-Microglia
	Microglia
	Oligodendrocytes
Alexander disease	Astrocytes		

## Funding

This work was supported by the National Center of Competence in Research (NNCR) “SYNAPSY” (n° 51AU40-125759), by FNRS grant 310030B-148169/1, and by the Préfargier and Panacée Foundations.

## Conflict of Interest Statement

The authors declare that the research was conducted in the absence of any commercial or financial relationships that could be construed as a potential conflict of interest.
